# Should We Turn off the Music? Music with Lyrics Interferes with Cognitive Tasks

**DOI:** 10.5334/joc.273

**Published:** 2023-05-04

**Authors:** Alessandra S. Souza, Luís Carlos Leal Barbosa

**Affiliations:** 1 Center for Psychology, Faculty of Psychology and Education Sciences, University of Porto, Porto, Portugal

**Keywords:** auditory distraction, lo-fi, judgments of learning, music, metacognition

## Abstract

People often listen to music while doing cognitive tasks. Yet, whether music harms or helps performance is still debated. Here, we assessed the objective and subjective effects of music with and without lyrics on four cognitive tasks. College students completed tasks of verbal and visual memory, reading comprehension, and arithmetic under three conditions: silence, instrumental music, and music with lyrics. Participants judged their learning during and after each condition. Music with lyrics hindered verbal memory, visual memory, and reading comprehension (*d* ≈ –0.3), whereas its negative effect (*d* = –.19) on arithmetic was not credible. Instrumental music (hip-hop lo-fi) did not credibly hinder or improve performance. Participants were aware of the detrimental impact of the lyrics. Instrumental music was, however, sometimes perceived as beneficial. Our results corroborate the general distracting effect of background music. However, faulty metacognition about music’s interfering effect cannot fully explain why students often listen to music while studying.

We listen to music, intentionally or not, while performing different activities (Rentfrow, 2012). In most tasks, music is a foreign stimulus, as when background music is playing while we are reading, studying, or solving mathematical problems. So far, both benefits and costs of background music on these tasks have been observed. Yet, people seem to have low insight into music’s potential impairing effects, often reporting actively choosing music as a background to study. We present further evidence that music with lyrics is generally detrimental to cognitive performance, while instrumental music has a more minor, not credible effect. People were usually aware of the distracting effect of the lyrics, yet they tended to believe that instrumental music was beneficial. Metacognition was, therefore, not wholly faulty, and may only partially explain inefficient study habits.

## Background Music: Why is the Evidence Mixed?

In their meta-analysis, Kämpfe et al. (2011) reported that the effect of background music on cognition was overall null because positive and negative results averaged each other out (see also De la Mora Velasco & Hirumi, 2020). There are several candidate variables to explain this variability. One potential variable is the presence of lyrics. A recent systematic review did not find a relation between the type of music and whether positive or negative effects were observed (de la Mora Velasco & Hirumi, 2020). Yet, a meta-analysis focused on reading observed a larger detrimental impact of music with lyrics than instrumental music (Vasilev et al., 2018). To further clarify this matter, we assessed the effects of both music with lyrics and instrumental music on cognitive performance.

Another potential moderating variable is the type of learning task. The most commonly used task is verbal memory, with the majority of studies finding negative effects in this task (Cassidy & MacDonald, 2007; Furnham & Bradley, 1997; Groot & Smedinga, 2014; Nittono, 1997; Reaves et al., 2016; Smith & Morris, 1977; Su & Wang, 2010). However, there are also reports of positive (de Groot, 2006) and neutral effects on this task (Jäncke et al., 2014; Jäncke & Sandmann, 2010; Küssner et al., 2016; Linek et al., 2011; Nguyen & Grahn, 2017). The use of visual tasks is rare, with only one study reporting worse visual memory when listening to music with lyrics compared to instrumental music (Belsham & Harman, 1977). Results for reading comprehension tasks are also mixed. Yet, in a recent meta-analysis, Vasilev et al. (2018) estimated an overall small but credible impairment of background music on reading (Hedges’s *g* = –0.19). Finally, fewer studies tested the effect of music on arithmetic tasks observing benefits (Miller & Schyb, 1989; Proverbio et al., 2018; Wolf & Weiner, 1972), no change (Chew et al., 2016; Manthei & Kelly, 1999; Mowsesian & Heyer, 1973; Wolfe, 1983), and even costs (Christopher & Shelton, 2017; Tucker & Bushman, 1991). The present study considered these multiple task domains to gain a clearer picture of the impact of background music on cognition.

Variability could also be due to sampling. Studies usually use between-subject designs with overall low sample sizes (mean *N* = 67), which afford less power to detect effects (de la Mora Velasco & Hirumi, 2020; Kämpfe et al., 2011). To more firmly establish the polarity and size of the effects of background music, we employed a within-subjects design with a relatively large sample.

Despite the mixed findings regarding the impact of background music on learning, students often report listening to music while studying or doing coursework. This raises the question regarding the subjective effect of music, or in other words, how people perceive the impact of music on their own performance.

## Metacognition and the Effects of Background Music

One recent survey estimated that people listen to music ca. 40% of the time while reading or writing, and 20% of the time while memorizing (Goltz & Sadakata, 2021). In other surveys, these values were of 60% while studying and 20% while reading (David et al., 2015; Kiss & Linnell, 2022). In general, participants were divided in assessing the impact of background music as costly or beneficial, but people that study with music tended to perceived it as beneficial (Goltz & Sadakata, 2021).

Few studies considered the subjective impact of music; but the general finding is that people have poor metacognition on this subject. Hallam and Godwin (2015) assessed the impact of calming *vs*. exciting music (as opposed to silence) on the quality of story-writing in children. Exciting music was detrimental to performance, yet this type of music was perceived as more enjoyable and hence as beneficial. Anderson and Fuller (2010) assessed reading comprehension in 7^th^ and 8^th^ graders in a musical and silence condition. Performance was lower in the musical condition, and this effect was larger for students that reported a preference for listening to music while studying. Christopher and Shelton (2017) asked participants to judge their performance in musical and silence conditions while performing reading and arithmetic tasks. Background music hindered performance in both tasks, but participants were unaware of its detrimental effect.

Overall, this scarce literature points to a metacognitive blind-spot: students seem to enjoy studying while listening to music, and hence they fail to perceive its true impact on performance. To offer more data on this subject, we asked participants to provide subjective assessments during and after completing our tasks.

## Present Study

Our goals were three-fold. First, we aimed to determine the effect of background music on four different cognitive domains: verbal and visual memory, reading comprehension, and arithmetic. Second, we contrasted performance in an instrumental music and music with lyrics condition to silence, using a within-subjects design and a relatively large sample of participants (*N* = 113–123). The inclusion of these two types of music permitted us to address the role of music type, while our sample-size provided a power of 80% to detect effects as low as *d* = .26. Third, we collected metacognitive judgments during and after the completions of our tasks to identify if people misperceive the impact of background music on their performance.

We formulated the following hypotheses. First, given that music with lyrics contains speech information, we expected this condition to create the largest interference in tasks that involve verbal processing. This prediction is based on the *Irrelevant Speech Effect*, namely the impairment of performance observed when irrelevant background speech is presented concurrently with a memory task (Colle & Welsh, 1976; LeCompte et al., 1997; Salamé & Baddeley, 1982, 2013). Our second prediction was that instrumental music would be less disruptive than music with lyrics. In the memory literature, background sounds were also found to disrupt performance (Jones & Macken, 1993). This prediction is however not without controversy. Instrumental music could also be predicted to produce better performance due to changes in emotional states, e.g., by relaxing participants (Kiss & Linnell, 2022).

Finally, we predicted that participants would show low metacognitive insight about the impact of background music on their performance. Given the scarce data on the literature, we had no specific prediction regarding an interaction with task or time-point in which the judgments were made (i.e., during or after the task).

## Method

### Participants

Psychology students from the University of Porto participated in this online study in exchange for extra-course credits. We choose to collect data online since it permitted the recruitment of a large sample of participants with reasonable data-quality (Uittenhove et al., 2023). The study protocol was approved by the Ethics Committee of the Faculty of Psychology and Education Sciences of the University of Porto (approval number 2022/02–05). Participants completed an Informed Consent Form online and were debriefed regarding the study purposes at the end.

The study was divided in two online sessions lasting 30 min each. We aimed to collect data of at least *N* = 100. Actual sample-size was determined based on participation sign-up. We accepted all submissions made during the spring semester of 2022 (from April to June). A total of 136 students participated, yet due to desistance or data-loss, we only obtained data-sets of both sessions of *N* = 100. Yet, to use as much data as possible, we considered all data we had for each of the tasks in isolation. Session 1 was completed by 123 students (*n* = 57 completed the verbal/math tasks; *n* = 66 completed the visual/reading tasks). Session 2 was completed by 113 students (*n* = 56 completed the verbal/math tasks; *n* = 57 completed the visual/reading tasks). Only 105 participants filled the demographics questionnaire at the end of Session 2. Respondents were aged between 18 and 58 years (*M* = 20.74, *SD* = 5.5), with 5% identifying themselves as “male”, 90.5% as “female”, 4% as “non-binary/third gender” and 1% as “rather not say”.

### Experimental Design

All variables were manipulated in a within-subjects design. Participants completed four tasks, with each task being completed three times in blocks that varied in terms of the background sound. In the *Silence* block, participants completed the task under normal ambient noise. Since the study was collected online, this reflected the usual ambient noise of the participants. We instructed participants to be in a quiet place, and to remove all possible distractions from the environment (TV, music, cell-phones, social media, animals, or other people). In the *Instrumental Music* block, participants listened to instrumental music while completing the task. We selected a genre of instrumental music known as lo-fi hip-hop due to its popularity among students for use while studying (Winston & Saywood, 2019). In the *Lyrical Music* block, participants listened to popular music with European Portuguese lyrics.

Before the start of the tasks in each session, participants were told to fetch a head-set or to be in an ambient were they could leave the computer sound enabled. Then they were instructed to adjust the volume to a comfortable level while listing to a sample sound. They were instructed to not change the volume level while working on the tasks. Before each task block, they were warned about the upcoming condition (silence, instrumental music, or music with lyrics). The order of the silence, instrumental, and lyrical blocks within each task was randomly determined for each participant and task.

### Materials

Participants completed four tasks: a verbal recall task, a visual recall task, a reading comprehension, and an arithmetic problem-solving task.

In the *verbal recall task*, participants learned three lists with 20 words each. One random word-list was assigned to be learned in each experimental condition. Words were drawn from the Minho word pool (Soares et al., 2017), which is a data-basis with 3,800 European-Portuguese words, ranked on imageability, concreteness and subjective frequency. Each of the three lists contained five words with high frequency (*M* = 189.7 per million; *SD* = 87.8) and high concreteness (*M* = 6.4; *SD* = 0.3), five words with high frequency (*M* = 289.5; *SD* = 233.3) and low concreteness (*M* = 2.7; *SD* = 0.3), five words with low frequency (*M* = 0.2; *SD* = 0.2) and high concreteness (*M* = 6.3; *SD* = 0.3), and five words with low frequency (*M* = 0.6; *SD* = 0.2) and low concreteness (*M* = 2.8; *SD* = 0.2). Additionally, four words were used as practice words. The word lists used are available in the Online Supplementary Materials.

For the *visual recall task*, three lists of 20 images were created, and one random list was assigned to be learned in each experimental condition. The images were selected from the ones used by Sutterer and Awh (2016). The solid color of each image was randomly sampled from 360 continuous colors selected from a color wheel defined in the CIELAB color space with the following parameters: L = 70, a = 20, b = 38, with a radius of 60 (Zhang & Luck, 2008). The color of each image remained the same for all participants. The images used and their colors are presented in the Online Supplementary Materials.

For the *reading comprehension task*, six lists of 20 sentences were constructed. Two lists were allocated to be processed in each experimental condition. These lists were made by adapting the Reading Test-Sentence Comprehension [Teste de Leitura: Compreensão de Sentenças (TELCS)], which is a Portuguese adaptation of the Lobrot’s Lecture 3 (L3) reading test (de Araújo Vilhena et al., 2016). Since the TELCS only contained 36 sentences, 84 additional sentences were created to obtain a total of 120 sentences. The TELCS is composed of a list of 36 incomplete sentences, where the last word is missing, for example, the sentence “*Foi difícil ter uma boa nota naquele*” [“It was hard to have a good grade on that”]. Participants are asked to select one word out of five available options to correctly complete the sentence. In the example above, the correct option was “*exame*” [“exam”]. The five options share at least one common characteristic. They can be visually similar by sharing a similar orthography and having letters in common with one another (e.g., “*enxada*”). They can be phonologically similar by sharing the same last phoneme and thus rhyming (e.g., “*enxame*”). Or they can be semantically proximal by having close meanings to each other (e.g., “*estudo*”). Using this model for the generation of the distractor words, the 84 sentences and their respective options were created. There was also an effort to make sure that the number of letters in the sentences did not differ much from list to list (the number of letters was between 880 and 980). The original sentences from the TELCS were distributed equally between the six lists and in order (e.g., sentence 1 in list 1, sentence 2 in list 2, etc.). The newly generated sentences were distributed equally between the lists. The sentence lists are presented in the Online Supplementary Materials.

For the *arithmetics task*, six lists consisting of 20 problems were constructed. Two lists were randomly assigned to be completed in each experimental condition. Each list contained six problems that followed the model “a × b + c” (e.g., 4 × 9 + 8 = ?), six problems that followed the model “a × b–c” (e.g., 7 × 5–3 = ?) and eight problems that followed the model “a + b × c” (e.g., 9 + 3 × 8 = ?). The list also contained five response options for each problem: the correct answer and four incorrect answers, which were generated by randomly assigning four numbers which were between the two closest multiples of 10 to the correct answer. For example, if the correct answer was 17, then the randomly generated wrong answers were between 10 and 20, for instance 14, 19, 11, 20. The operation lists are presented in Online Supplementary Materials.

Each task was completed in three separate blocks, each representing a different experimental condition, with music being presented concurrently in two of them. Therefore, a total of eight songs were chosen to be presented across the four tasks. Four of the eight songs contained only lo-fi instrumental music. They were retrieved from the Youtube channel “Lofi Girl” which had 10,2 million subscribers and 1 165 531 540 overall views on 23/03/2022. Its most viewed video had 75 688 049 views and it consists of a compilation of 28 songs. Using a random number generator, four numbers were randomly chosen from 1 to 28 (3, 25, 17 and 2). The songs corresponding to each of these positions were chosen, which were: “Cotton Cloud” by Fatb; “Gyoza” by less.people; “Alone Time” by Purrple Cat; “Snowman” by WYS. The remaining four songs were popular songs with lyrics in European Portuguese. Using the website Acharts.co, we consulted which were the most popular songs in Portugal on 20/02/2022, and then chose the four most popular ones. The first four songs which followed our criterion were at the spots 13, 14, 26, and 28. The songs were: “Onde Vais” by Bárbara Bandeira e Carminho; “Mais ou Menos Isto” by Rita Rocha; “Fato treino do City” by Sippinpurpp; “Como Se Te Fosse Perder” by Anselmo Ralph e Diogo Piçarra. The pairing of each task and song was the same for all participants.

### Procedure

All tasks were designed to be completed online. Tasks were programmed using the free and open source lab.js online experimenter builder (Henninger et al., 2020, 2022). This builder uses HTML and Java-script as the programming language. The code to run all tasks is available at: https://osf.io/xcv6e/.

The experiment was divided into two sessions lasting ca. 30 min each, which were completed in separate days. On one session, participants completed the verbal memory recall and the arithmetic tasks. On the other session, they completed the visual recall and the reading comprehension tasks. This was done to assure minimal interference between the tasks. The order of tasks within each session was randomly determined for each participant. We counterbalanced the order of the sessions. Half of the participants completed the verbal recall and arithmetic tasks on the first session, and the visual recall and reading comprehension tasks in the second session. For the remaining ones, the order was reversed.

In the first session, participants were presented an informed consent describing the study. Participants were asked to consent to the terms of the experiment before advancing to the task. Next, participants created an individual code to connect the data of the two online sessions. Then, participants were guided to complete a volume test so that they could adjust the volume on their computer to a comfortable level before proceeding. They were advised to not change the volume throughout the whole experiment. Participants were then instructed on the completion of each of the two tasks to be carried out in that session. At the end of the first session, they received a certificate of completion of the task, and were instructed to contact the experimenter to receive the link to the second session.

The sequence of events in Session 2 was similar with three major differences: (1) it did not contain the informed consent form; (2) at the end of the session, participants completed demographic questions (age, gender, schooling levels of the participant and the participant’s parents) and questions about their study habits (i.e., their average daily study hours, how frequently they listen to music while studying and how frequently they study in noisy places); and (3) it contained a debriefing about the experiment at the end.

**Figure 1:**
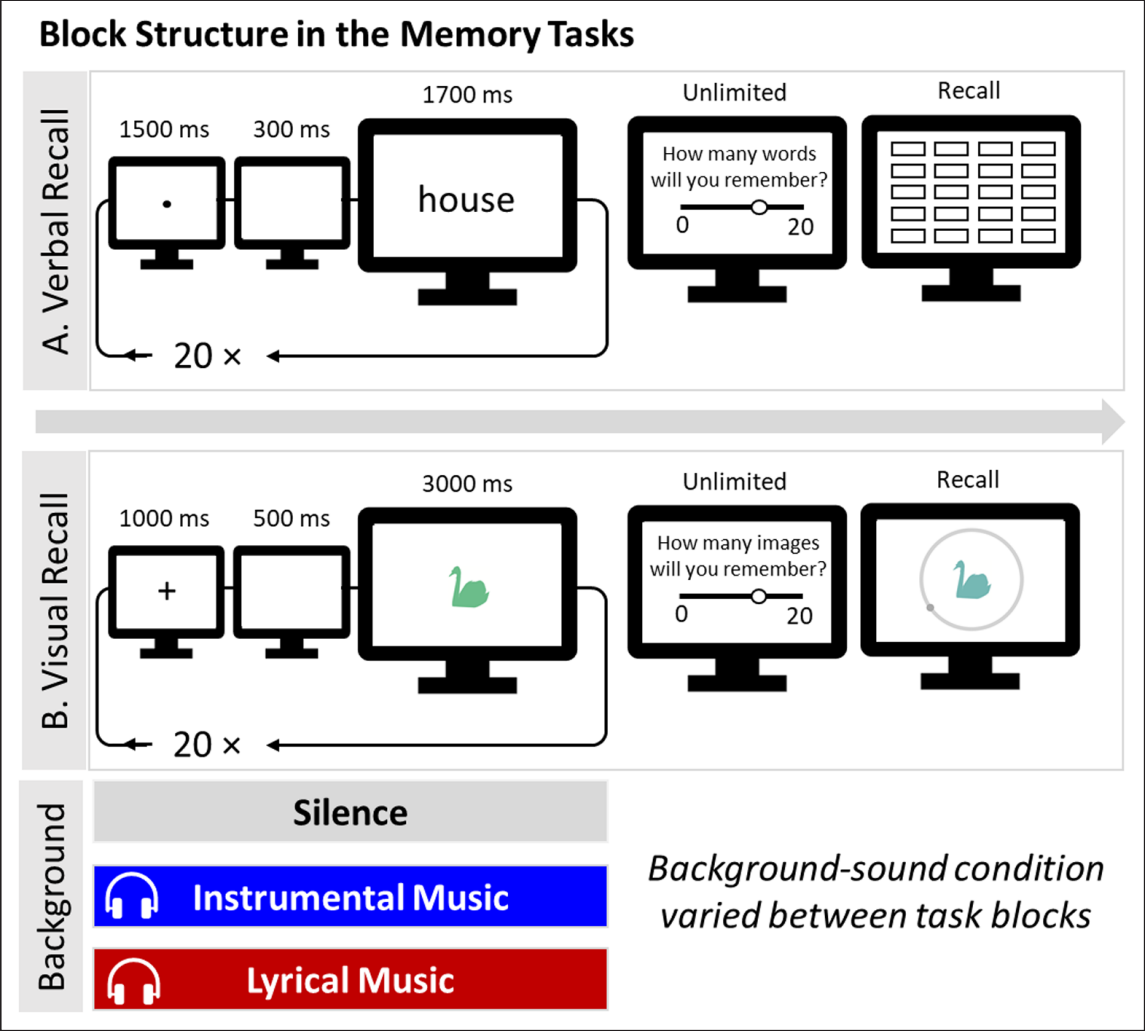
Illustration of the Flow of Events in Word and Visual Recall Tasks. *Note*: Each task was completed three times, once in silence, once while listening to instrumental music (lo-fi) and once with music with lyrics. Background music was presented during the study phase only.

All four tasks began with an introductory instruction and an example trial. In the *word recall task*, each task block consisted of the presentation of a sequence of 20 words in the center of the screen, one-by-one, for memorization. As shown in Figure 1A, before each word, a fixation point appeared in the middle of the screen for 1.5 s, followed by a blank screen for 0.3 s. Afterwards, the word was presented for 1.7 s. In the music blocks, music was presented only while learning the memoranda. After all words were displayed, participants were asked about the number of words they will be able to recall (aka a judgement of learning). They answered by moving a slider ranging from 0 to 20. Then the recall phase began: participants were instructed to recall as many of the memorized words as they could in any order. They were shown a grid with 20 cells. Every time they entered a word followed by enter, the cursor moved to the next cell. When they were finished recalling the words, they clicked on a “finish” button at the bottom of the page to move on. They could recall between 0 and 20 words, with no time restriction. Once they entered a word they could not edit their response. After finishing the recall, participants rated the difficulties they felt in the memorization and in the recall part by using two sliders that ranged from 0= “Little difficulty” to 10 = “A lot of difficulty”.

For each block, one of the three word-lists created for this task was randomly used (see Materials section). After the completion of all blocks in this task, participants completed a series of follow-up questions regarding the manipulation of background music. First, they were asked to rate how much the two types of music affected their performance compared to performance on the silence block. They answered using two sliders (one for the instrumental and one for the music with lyrics condition) that ranged from –10 (“Very negatively”) to 10 (“Very positively”). Next, participants were asked to provide some information in relation to the songs: if they knew the songs; how much they enjoyed the instrumental music from 0 to 10; and the music with lyrics from 0 to 10.

In each block of the *visual recall task*, a sequence of 20 colored images were displayed on the center of the screen one-by-one. As shown in Figure 1B, before each image, a fixation cross appeared on the middle of the screen for 1 s, followed by a blank screen for 0.5 s. Afterwards, the colored image was presented for 3 s. After all images were displayed, the participant was asked to make a judgment of learning, similarly to the *verbal recall task*. Then the recall phase began, in which all 20 images were probed in random order. Each image was presented first in gray color surrounded by a gray wheel. When the participant hovered the cursor over the grey wheel, the color of the image changed continuously. This is because the grey wheel was covering a continuous color wheel. By moving the mouse around the grey wheel, the participant continuously adjusted the color of the probed image. Participants were instructed to click with the mouse when they thought they had selected the correct color. There was no time-limit to respond in the recall phase. Thereafter, the next to-be-recalled image was presented. Between images, a fixation point appeared for 1 s. When they were finished recalling all images, the next block started. After the completion of all blocks in this task, participants completed the same follow-up questions regarding the manipulation of music as described for the verbal recall task.

**Figure 2:**
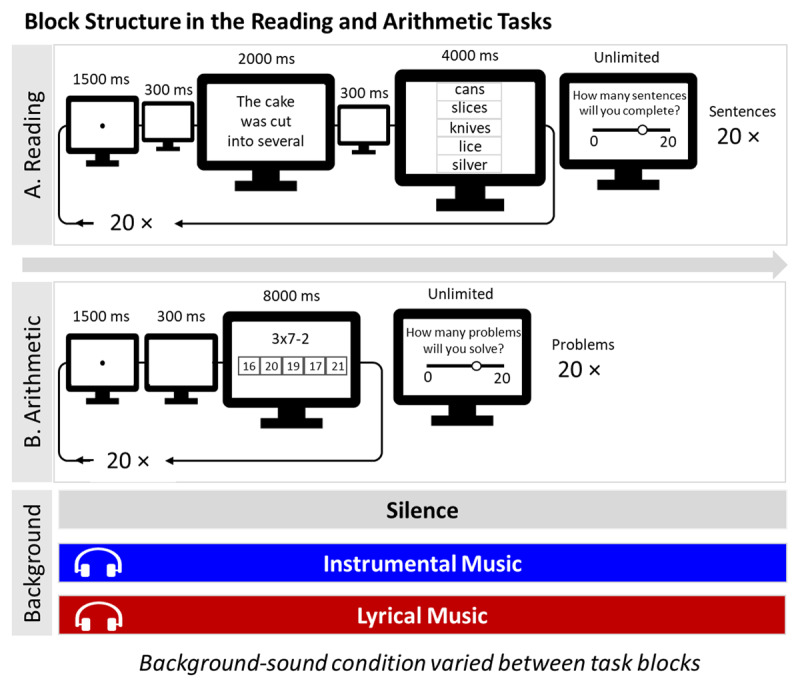
Illustration of the Flow of Events in the Reading Comprehension and the Arithmetic Tasks. *Note*: Each task was completed three times, once in silence, once while listening to instrumental music (lo-fi) and once with music with lyrics. Background music was presented during completion of the task and the judment of learning rating.

Each block of the *reading comprehension task* was divided into two parts, separated by a judgement of learning. Each part consisted of the presentation of a sequence of 20 sentences (i.e., one of the lists generated for this task). As illustrated in Figure 2A, each sentence was preceded by a fixation point (1.5 s), followed by a blank screen (0.3 s). Afterwards, a sentence appeared on the center of the screen for 2 s. A blank screen appeared once more for 0.3 s before the five options were displayed for 4 s. The five options were randomly contained inside rectangles, all centered and positioned vertically in the center of the screen. Participants had to click on the option they thought completed the sentence correctly. If they did not respond within 4 s, a time-out was registered and the program moved to the next event. Time-outs were counted as incorrect answers. After the first 20 sentences, participants were asked to make a judgment of learning by predicting how many sentences they would complete correctly in the next half of the block. They answered by moving a slider ranging from 0 to 20. Then, the next 20 sentences were presented. It followed the same structure as the first one. In blocks in which background music was played, the music was continuously looping while participants completed both block parts and the judgment of learning rating. For each participant, the six sentence-lists created for this task were randomly distributed across the silence, instrumental and lyrical blocks, and the two task parts therein. After the completion of all blocks in this task, participants completed the same follow-up questions regarding the manipulation of background music as detailed previously.

The blocks of the *arithmetic task* followed a similar structure to the ones in the *reading comprehension task*. Each block was divided into two parts, each consisting of the presentation of a sequence of 20 problems (see Figure 2B). Each problem was anticipated by a fixation point (1.5 s), followed by a blank screen (0.3 s). Afterwards, a problem and the respective response options appeared simultaneously for 8 s. For each participant, the five options were randomly displayed inside five rectangles, all centered and positioned along the horizontal axis in the middle of the screen. Participants clicked on the option they thought was the correct answer to the problem. If they did not respond within 8 s, a time-out was registered and the programed moved to the next event. After the first 20 problems, participants were asked to make a judgment of learning by predicting how many problems they believed they would solve correctly in the next block half. They answered by moving a slider ranging from 0 to 20. Next, the final sequence of 20 problems followed. In blocks with background music, the music was presented continuously through the presentation of the problems and the judgment of learning. For each participant, the six problem-lists created for this task were randomly distributed across the three condition blocks (i.e., silence, instrumental, and lyrical), and block half. After the completion of all blocks, participants answered the follow-up questions regarding the manipulation of music.

### Data Analysis

The data was processed and analyzed using R (R Core Team, 2021) and Rstudio (Rstudio Team, 2020). Statistical inferences were performed using the BayesFactor package (Morey & Rouder, 2015) using the default prior settings. We used Bayesian Inference to assess the evidence for the presence vs. absence for the effect of background music on performance. In essence, Bayesian inference provides a comparison of the likelihood of the data in light of the alternative hypothesis (i.e., there is an effect of a manipulation) and the null hypothesis (i.e., no effect). The ratio of the likelihood of these hypotheses is the Bayes Factor (BF). Here we report BF_10_, which represents the strength of the evidence for the alternative hypothesis over the null. BFs should be interpreted as a continuous measure. BF_10_ > 1 provides evidence for the alternative hypothesis, and BF_10_ < 1 provides evidence for the null hypothesis. For example, a BF_10_ = 10 indicates that the data is 10 times more likely under the alternative hypothesis than the null. Conversely, a BF_10_ = 0.10 indicates that the data is 10 times more likely under the null than the alternative hypothesis.

The dependent variables in our study varied depending on the task. The following objective measures of performance were considered. For the verbal recall task, we computed the proportion of correctly recalled words over the maximum number of words learned (i.e., 20). For the visual recall task, first, we computed a measure of recall error (Souza et al., 2014). Recall error reflects the absolute distance on the wheel between the angle of the correct response and the angle of the response given by the participant. For example, if the correct color was at the angle 30°, and the participant recalled the color at the angle 57°, the recall error was of 27° in that trial. The measure of recall error ranges from 0° (perfect recall) to 180° (recall of the color at the opposite location on the wheel). An average performance close to 90° is consistent with guessing. To increase comparability with the remaining measures, we rescaled the recall error variable to range between 0 and 1 (as in proportion correct), with larger values reflecting better performance. We applied the following equation: (180-recallError)/180. In this rescaled measure, 0.5 indicates guessing, and 1.0, perfect recall. For the reading comprehension and arithmetic tasks, we computed two measures: the proportion of correct responses (with time-outs being considered as wrong responses), and the time to respond correctly in the task (in seconds) since the onset of the response options. The maximum response time in the reading and arithmetic tasks was 4 and 8 s, respectively.

We collected two subjective measures. Judgments of learning were requested in all four tasks and reflected the predictions of performance during the task. The predictions were made in absolute values (e.g., *how many words do you think you will remember?*) and they were transformed in proportions by dividing it by the maximum value (i.e., 20 in all tasks). Finally, participants also made post-task ratings of how much they believed the two types of musical background affected their performance in comparison to the silence condition (from very negatively = –10 to very positively = 10).

### Research Transparency and Openness

All task materials, data, and analysis scripts are available in the page of the project at the Open Science Framework: https://osf.io/xcv6e/ (Souza & Barbosa, 2023).

## Results

### Outlier Detection

We first screened the data for potential outliers. For the reading comprehension and arithmetic tasks, we calculated the 99^th^ quantile of a binomial distribution with 40 trials (i.e., the number of trials per condition in each task) and with a probability of success of 0.2 on each trial (one correct option out of five), and divided this by the total number of trials. This cutoff value (0.35) indicates the level of performance that is no better than chance. Then, we excluded participants with an overall level (i.e., across all conditions) of correct responses smaller or equal than this cutoff. No outliers were found in the reading comprehension task (*N* = 123), but seven were excluded in the arithmetic task (final *N* = 106). For the visual recall task, a similar method was used, but using an average recall error of 80° as the cutoff point instead. In this task, random responding is assumed to generate responses close to 90°. Five outliers were removed (final *N* = 118). In the verbal recall task, we simply calculated the proportion of correct recalled words, and since this task does not have a guessing level, we included all respondents (*N* = 113).

Table 1 presents the evidence for the main effect of background music in the objective performance measures (i.e., proportion correct and time to respond) and in the subjective measures of performance (i.e., judgments of learning and post-task ratings) in all four tasks.

**Table 1:** Evidence (Bayes Factor, BF) for the Main Effect of Background Music (One-Way ANOVA), and for the Pairwise Contrast of Conditions (t-tests). For the Condition Comparisons, the Effect-Size (Cohen’s d) and its 95% Confidence Interval is Also Provided.


TASK	DEPENDENT VARIABLE	MUSIC BF_10_	PAIRWISE CONDITION CONTRASTS		

			INSTRUMENTAL *VS.* SILENCE	LYRICAL *VS.* SILENCE	INSTRUMENTAL *VS.* LYRICAL

Verbal Recall	Proportion Correct	11.44	BF_10_ = 0.33 *d* = –.16 [–.34, .03]	BF_10_ = 20.43 *d* = –.32 [–.51, –.13]	BF_10_ = 0.84 *d* = .20 [.01, .38]

	Judgment of Learning	1.07 × 10^7^	BF_10_ = 0.11 *d* = –.01 [–.20, .17]	BF_10_ = 74.38 *d* = –.36 [–.55, –.17]	BF_10_ = 5.39 *d* = .27 [.08, .46]

	Post-Task Rating		BF_10_ = .11 *d* = –.02 [–.20, .17]	BF_10_ = 1.68 × 10^7^ *d* = –.64 [–.84, –.44]	

Visual Recall	Proportion Correct	24.6	BF_10_ = 1.85 *d* = –.23 [–.41, –.04]	BF_10_ = 40.79 *d* = –.33 [–.52, –.15]	BF_10_ = 0.32 *d* = .14 [–.04, .32]

	Judgment of Learning	77.25	BF_10_ = 0.96 *d* = –.20 [–.38, –.02]	BF_10_ = 92.92 *d* = –.35 [–.53, –.16]	BF_10_ = 1.06 *d* = .20 [.02, .38]

	Post-Task Rating		BF_10_ = 14.24 *d* = .29 [.11, .47]	BF_10_ = 64.13 *d* = –.34 [–.52, –.15]	

Reading	Proportion Correct	8.73	BF_10_ = 0.29 *d* = .14 [–.05, .32]	BF_10_ = 0.70 *d* = –.19 [–.37, .00]	BF_10_ = 18.25 *d* = .31 [.12, .50]

	Time to Respond	0.17	BF_10_ = 0.12 *d* = –.52 [–.23, .14]	BF_10_ = 0.53 *d* = –.17 [–.36, .01]	BF_10_ = 0.24 *d* = .13 [–.06, .31]

	Judgment of Learning	506.25	BF_10_ = 0.49 *d* = –.17 [–.35, .02]	BF_10_ = 465 *d* = –.40 [–.60, –.21]	BF_10_ = 9.66 *d* = .29 [.10, .48]

	Post-Task Rating		BF_10_ = 3.08 × 10^5^ *d* = .53 [.34, .72]	BF_10_ = 4.22 *d* = –.25 [–.43, –.07]	

Arithmetic	Proportion Correct	0.067	BF_10_ = 0.12 *d* = –.05 [–.24, .14]	BF_10_ = 0.21 *d* = –.11 [–.31, .08]	BF_10_ = 0.14 *d* = .07 [–.12, .26]

	Time to Respond	0.041	BF_10_ = 0.11 *d* = –.02 [–.21, .17]	BF_10_ = 0.12 *d* = .04 [–.15, .23]	BF_10_ = 0.12 *d* = .05 [–.14, .24]

	Judgment of Learning	1.71	BF_10_ = 0.99 *d* = –.21 [–.40, –.02]	BF_10_ = 2.32 *d* = –.25 [–.44, –.05]	BF_10_ = 0.20 *d* = .11 [–.08, .30]

	Post-Task Rating		BF_10_ = 0.15 *d* = .08 [–.11, .27]	BF_10_ = 3.45 × 10^6^ *d* = –.63 [–.84, –.42]	


*Note*. Positive values of *d* reflect better performance of the musical condition stated first; negative values reflect worse performance in this condition.

### Verbal Recall

**Figure 3:**
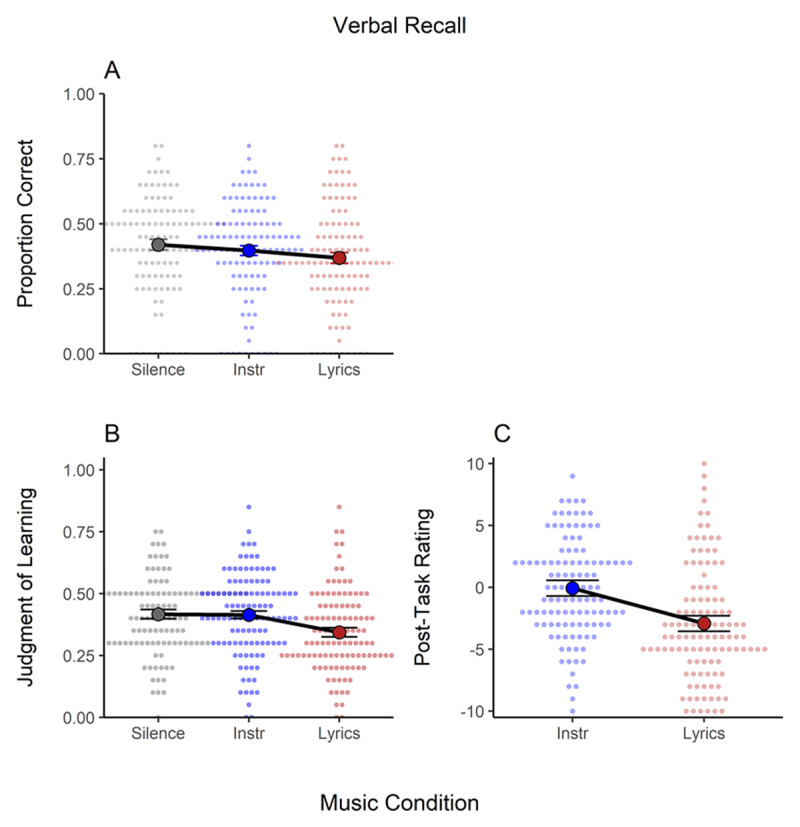
Results of the Verbal Recall Task. Panel A. Proportion of Correct Answers. Panel B. Judgments of Learning. Panel C. Post-Task Ratings. *Note*: Instr. = instrumental music. Individual data is shown as a small overlaid cloud of dots (slightly jittered along the x-axis for better visibility). The sample mean is presented as a large dot. Error bars are the 95% within-subject confidence interval (Morey, 2008).

Figure 3 presents the mean proportion of correct answers (Panel A), judgements of learning (Panel B), and post-task ratings (Panel C) in the verbal recall task. Background music credibly affected proportion correct (see Table 1), this being mainly due to a small decrease in recall accuracy (*d* = –.32) in the lyrical condition compared to silence. The instrumental condition produced a smaller (*d* = –.16) decrement, which made this condition not credibly different from either the silence or lyrical conditions. Judgments of learning showed a similar pattern, with participants predicting lower performance in the lyrical than in the remaining two conditions, which did not differ. At the end of the task, participants accurately evaluated instrumental music as having no credible effect compared to silence (value close to 0), but lyrical music as leading to a cost (value < 0).

### Visual Recall

**Figure 4:**
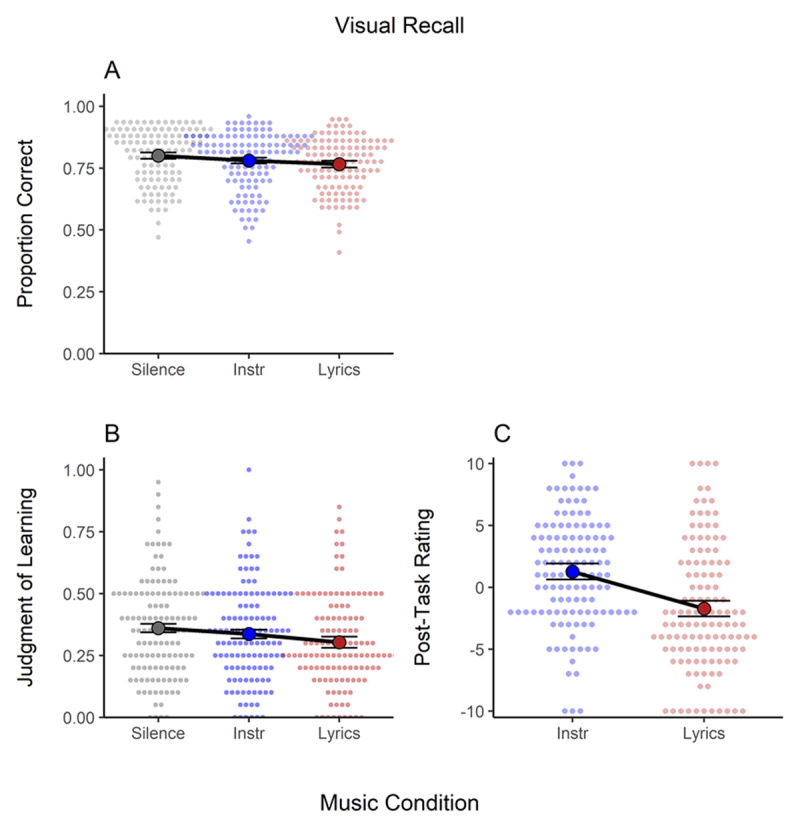
Results of the Visual Recall Task. Panel **A**. Proportion of Correct Answers. Panel **B**. Judgments of Learning. Panel **C**. Post-Task Rating. *Note*: Instr. = instrumental music. Individual data is shown as a small overlaid cloud of dots (slightly jittered along the x-axis for better visibility). The sample mean is presented as a large dot. Error bars are the 95% within-subject confidence interval (Morey, 2008).

Figure 4 shows the proportion of correct responses (Panel A), judgments of learning (Panel B), and post-task ratings (Panel C) in the visual recall task. There was strong evidence *for* an effect of background music in visual recall (see Table 1). This was mainly due to a small decrease (*d* = –.33) in recall accuracy in the lyrical condition compared to silence. The effect of instrumental music was also negative (*d* = –.23), but it was ambiguous. Subjective measures also indicated that participants were aware of the detrimental impact of music with lyrics on their performance – both when they rated learning during the task as well as in the post-task ratings. In contrast, participants predicted similar performance in the instrumental condition as in the silence condition when asked during the task (i.e., judgments of learning, Panel B), but not in the post-task evaluations (Panel C) in which they considered instrumental music as beneficial to performance (values > 0).

### Reading Comprehension

**Figure 5:**
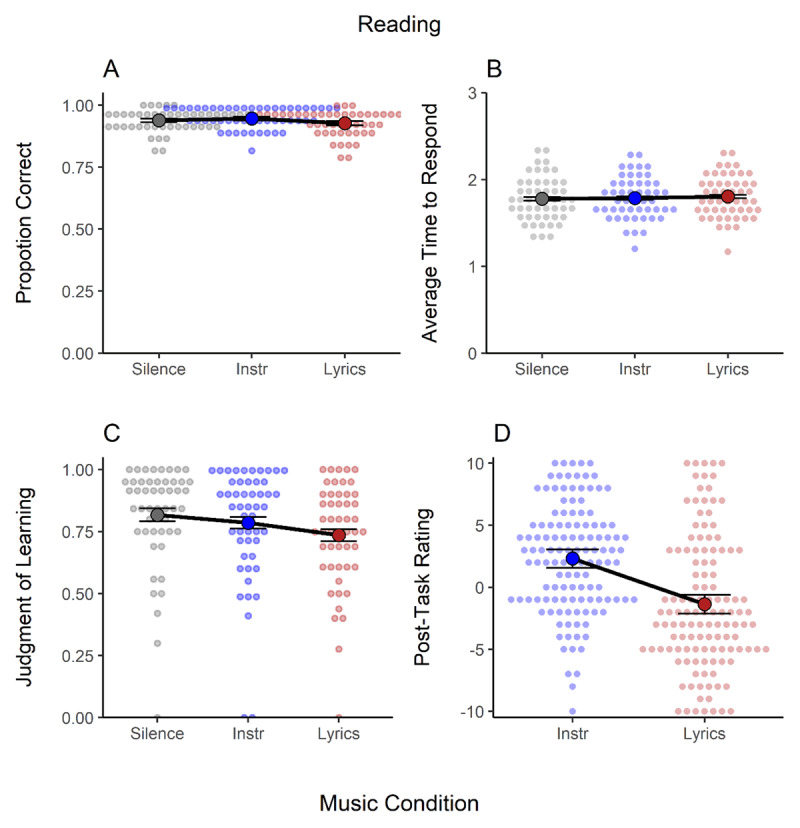
Results of the Reading Comprehension Task. Panel **A**. Proportion of Correct Answers. Panel **B**. Average Time to Respond Correctly. Panel **C**. Judgments of Learning. Panel **D**. Post-Task Ratings. *Note*: Instr. = instrumental music. Individual data is shown as a small overlaid cloud of dots (slightly jittered along the x-axis for better visibility). The sample mean is presented as a large dot. Error bars are the 95% within-subject confidence intervals (Morey, 2008).

Figure 5 presents the mean proportion of correct answers (Panel A), the average time to respond correctly (Panel B), judgments of learning (Panel C), and the post-task rating (Panel D) in the reading comprehension task. Although performance was generally very high in this task, there was substantial evidence for a music effect on the proportion of correct responses (see Table 1). This was due to somewhat higher accuracy in the instrumental (*M* = 0.95, *SD* = 0.22) compared to the lyrical condition (*M* = 0.93, *SD* = 0.26). It is worth noting that the contrast between the lyrical and silence conditions produced a small performance decrement (*d* = –.19) of the same size as reported in a recent meta-analysis (Vasilev et al., 2018). Another unique aspect is that this is the only task in which instrumental music tended to improve performance, but note that this effect was not credible. There was no effect on the time to respond correctly in the task.

With regard to the subjective measures of performance, during the completion of the task, judgments of learning (Figure 5C) were not credibly different between the instrumental and silence conditions, but were worse in the lyrical condition. In the post-task ratings (Figure 5D), however, participants judged instrumental music as beneficial (values > 0), whereas lyrical music was perceived as detrimental (values < 0) compared to silence.

### Arithmetic Task

**Figure 6:**
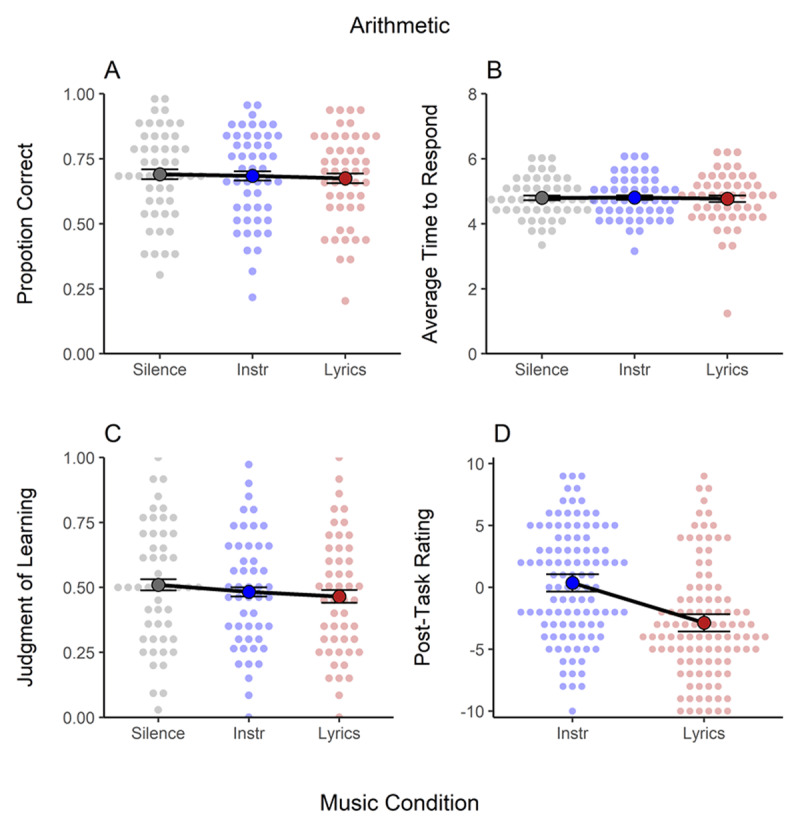
Results of the Arithmetic Task. Panel A. Proportion of Correct Answers. Panel B. Average Time to Respond Correctly. Panel C. Judgments of Learning. Panel D. Post-Task Ratings. *Note*: Instr. = instrumental music. Individual data is shown as a small overlaid cloud of dots (slightly jittered along the x-axis for better visibility). The sample mean is presented as a large dot. Error bars are the 95% within-subject confidence interval (Morey, 2008).

Figure 6 shows the proportion of correct responses (Panel A), time to respond correctly (Panel B), judgments of learning (Panel C), and post-task rating (Panel D) in the arithmetic task. There was strong evidence *against* an effect of background music in objective measures of performance (i.e., proportion correct and time to respond; see Table 1). Yet, when considering the proportion correct measure, the pattern was the same as in the previous tasks, with a slightly larger impairment for lyrical (*d* = –.11) than instrumental music (*d* = –.05) compared to silence. In Judgments of learning (Figure 6C), there was ambiguous evidence for an effect, mainly due to somewhat lower predictions in the lyrical condition compared to silence. In the post-task ratings (Figure 6D), participants accurately predicted that instrumental music was inconsequential to performance, but overestimated the detrimental impact of lyrical music.

### Music Preferences and Study Habits

After completing all three blocks of each task, participants were asked whether they knew each song, and how much they liked each song on a scale ranging from 0 (“*I didn’t like it*”) to 10 (“*I liked it a lot*”). For the verbal recall task, both songs were equally liked (instrumental: *M* = 5.50, *SD* = 2.64; lyrical: *M* = 5.23, *SD* = 3.36), BF_10_ = 0.13. For the visual recall task, the song with lyrics was liked slightly more (instrumental: *M* = 5.45, *SD* = 2.81; lyrical: *M* = 6.27, *SD* = 3.2) but this difference was ambiguous, BF_10_ = 1.29. For reading comprehension, the instrumental music (*M* = 6.04, *SD* = 2.70) was substantially more liked than the one with lyrics (*M* = 3.67, *SD* = 3.47), BF_10_ = 1.6×10^5^. Finally, for the arithmetic task, the instrumental music (*M* = 5.78, *SD* = 2.66) was also more liked than the one with lyrics (*M* = 4.18, *SD* = 3.31), BF_10_ = 153.98.

Participants were also asked “*Did you know any of the songs that were played?*”. They could answer by selecting one of four options: “*Yes, both*”, “*No, neither*”, “*Yes, but only the instrumental song*” and “*Yes, but only the song with lyrics*”. Table 2 presents song knowledge by task. In the recall tasks, the majority of participants knew the song with lyrics, whereas in the reading comprehension and arithmetic tasks the majority of participants was not familiar with neither song. Throughout all tasks, the instrumental song (lo-fi) was the least recognized one.

**Table 2:** Music Knowledge of Each Song in the Different Tasks.


TASK	SONGS	MUSIC KNOWLEDGE	FREQ.	%

Verbal recall		Both	8	7.1

		Neither	34	30.1

	*“Cotton Cloud”*	Only Instrumental	2	1.8

	*“Onde Vais”*	Only Lyrical	69	61.1

		Total	113	100

Visual recall		Both	8	6.5

		Neither	53	42.7

	*“Gyoza”*	Only Instrumental	1	0.8

	*“Mais ou Menos Isto”*	Only Lyrical	62	50

		Total	124	100

Reading Comprehension		Both	3	2.4

		Neither	84	67.7

	*“Alone Time”*	Only Instrumental	7	5.6

	*“Fato treino do City”*	Only Lyrical	30	24.2

		Total	124	100

Arithmetic		Both	5	4.4

		Neither	68	60.2

	*“Snowman”*	Only Instrumental	10	8.8

	*“Como Se Te Fosse Perder”*	Only Lyrical	30	26.5

		Total	113	100


Regarding study habits, participants reported studying for an average of three hours daily (*M* = 3.11, *SD* = 2.6). When asked how often they study while listening to music, 14.29% said always, 20% said often, 22.86% said sometimes, 24.76% said rarely and 18.1% said never. In a similar question, participants were asked how often they study in a noisy environment, to which 0% replied always, 5.71% replied often, 29.52% replied sometimes, 43.81% replied rarely and 20.95% replied never. This indicates that a noisy environment is a less common context of study for our participants than a musical environment.

## Discussion

We assessed the objective and subjective impact of background music on four cognitive tasks. Verbal and visual memory were significantly worse when these tasks were completed with music with lyrics compared to silence. In the reading comprehension task, participants responded more correctly in the instrumental than in the lyrical condition. Only in the arithmetic task, background music had no credible effect. Whereas music with lyrics was generally detrimental, our instrumental music (lo-fi) did not credibly hinder or improve performance. Subjectively, the music with lyrics was always perceived as impairing, even when it did not credibly hinder performance. Instrumental music, in contrast, was not seen as distracting, and retrospectively, participants tended to assess it as beneficial.

### Hypotheses of the Impact of Music on Learning

Based on the *Irrelevant Speech and Irrelevant Sound Effects*, we hypothesized a graded negative impact of background music: music with lyrics should be the most impairing, whereas instrumental music should lay in-between the lyrical and silence conditions. As shown in Table 1, our results generally agreed with these predictions. Music with lyrics had a credible, but relatively small effect (*d* = ca. 0.3) in three of our tasks. For arithmetic, the negative impact was smaller (*d* = –.19) and not credible, however, the direction of the effect was the same as in the remaining tasks. In contrast to silence, instrumental music had a much smaller impact on performance (*d*s ranging from –.23 to .14), which could not be credibly determined. There are two possible reasons for this. First, the impact of music with lyrics was already small, and given the even smaller interference produced by instrumental music, there was not much room to measure their difference. Second, our sample-size was not powered to find very small effects. Larger sample-sizes will be required to firmly establish if instrumental music harms performance or whether it is inconsequential.

Our findings agree with previous studies in indicating that background music has a general distracting effect (de la Mora Velasco & Hirumi, 2020; Kämpfe et al., 2011; Vasilev et al., 2018), and that the size of this distracting effect is moderated by music type. Music with lyrics contains speech, which has privileged access to our cognition. Although our study did not control that the lyrical and instrumental conditions differed only in terms of speech presence, a recent study showed that speech was the determinant variable in generating a distraction effect in a continuous reading task (Vasilev et al., 2022). Our results corroborate this assumption. This speech-related effect may be due to either semantic or phonological interference with the ongoing task (Vasilev et al., 2022).

Our instrumental music (hip-hop lo-fi) had a much milder, and not credible performance effect. This new musical genre is becoming popular among students, being generally advertised as music to listen while studying (*Lo-Fi Study Music*, n.d.; “The Benefits of Studying to Lo-Fi Music,” 2021; Winston & Saywood, 2019). Although lo-fi is sometimes referred to as a study booster, our findings lend little support for this claim. The only task in which lo-fi tended to improve performance (although not credibly) was reading comprehension. In the remaining tasks, it had the same negative trend as music with lyrics, only smaller. Our results therefore suggest that given the choice between music with lyrics vs. instrumental music to study to, instrumental music should be preferred. Yet, based on performance indicators alone, this condition should not be recommended or preferred over silence.

### Differences Between Task Domains

We assessed the impact of background music on a set of four cognitive tasks spanning different domains to assess if task type moderates the effect of background music. We employed standard memory tasks from the literature on verbal and visual memory. For reading comprehension and arithmetic problem solving, we adapted tasks to allow for a more continuous measure of processing through the online recording of accuracy and response times.

We obtained a reasonably consistent pattern across tasks: performance was generally best under silence, intermediate in the instrumental condition, and worse in the lyrical condition. Hence, we have little evidence that task type moderates the cognitive effects of background music. Reading comprehension slightly deviated from this pattern due to the somewhat performance increase in the instrumental condition. This was also the easiest task (accuracy > 90%). We adapted a reading test for online data-collection, enforcing short processing times in attempt to make the task more challenging. Yet, it was still quite easy for our sample of college students. Future studies may consider creating sentences with a more challenging structure to reduce ceiling effects.

In general, we expected lyrical music to have a more pronounced negative effect on verbal tasks. Yet, the general trend was the same for all tasks. Although it may seem surprising that visual memory was similarly impacted as verbal memory by music with lyrics, recent evidence is mounting that visuospatial tasks tend to be impaired by verbal as well as non-verbal means (Morey, 2018). Furthermore, presentation times in our visual task were sufficiently long to allow for participants to try to verbaly label the memoranda, and previous research from our lab has shown that labeling can improve episodic long-term memory (Overkott & Souza, 2022). Music with lyrics may therefore interfere with this labeling process, thereby harming performance.

### Awareness of the Impact of Music

Our second main aim was to assess awareness of the effect of music. Based on the previous literature (Christopher & Shelton, 2017; Hallam & Godwin, 2015), we hypothesized that metacognition would be low. Surprisingly, participants were quite aware of the negative impact of music with lyrics on performance. For all tasks, music with lyrics was perceived as distracting, even when it did not credibly hinder performance (arithmetic task). On the converse, instrumental music was perceived as not distracting. Perhaps the contrasting effect to the music with lyrics was so large that after completing the tasks, participants even assessed instrumental music as beneficial.

Our study is one of the first to chart subjective perceptions of the impact of music during and after cognitive tasks, ad to show that metacognition about the interfering impact of music was not faulty. Yet, the puzzle remains regarding why students often report listening to music while studying. Future studies may need to include not only subjective assessments of performance success (as done here) but also emotional and motivational effects of music listening. It is possible that cognitive, motivational, and emotional variables drive subjective experiences with music that may conjointly guide the self-regulation of study habits.

## Data Accessibility Statement

Materials, data, and analysis code can be accessed at: https://osf.io/xcv6e/.
